# Breeding SCN-resistant soybean lines with improved oil and protein contents

**DOI:** 10.3389/fpls.2025.1539409

**Published:** 2025-03-06

**Authors:** Yun Lian, Chenfang Lei, Dongwei Han, Ming Yuan, Weiguo Lu

**Affiliations:** ^1^ Institute of Crop Molecular Breeding, Henan Academy of Agricultural Sciences, Zhengzhou, China; ^2^ Institute of Soybean Research, Qiqihar Branch of Heilongjiang Academy of Agricultural Sciences, Qiqihar, Heilongjiang, China

**Keywords:** soybean, soybean cyst nematode, germplasm, oil, protein

## Abstract

Soybean cyst nematode (SCN) disease caused by *Heterodera glycines* is one of the most devastating diseases in soybean (*Glycine max* L.) and results in significant yield losses annually worldwide. Breeding crops for resistance is an eco-friendly approach to minimize these losses. In this study, SCN-resistant germplasm with excellent agronomic traits was obtained through cross-breeding between resistant germplasm sources and local cultivars that were high-yielding but susceptible to SCN in China’s two major soybean-growing regions. Using the single seed descent method, plants with favorable agronomic traits were selected and then planted into rows to raise seeds for marker assays and environmental-controlled bioassays. Finally, three lines from Northeast China and three from the Huang-Huai Valleys, all harboring resistance to races 1, 3, and 5, were selected. Their 100-seed weight ranged from 18.91 g to 21.6 g. The average oil contents of the three high-oil-content lines (QingF6-67, QingF6-98, and QingF6-99) from Northeast China ranged from 19.96% to 22.74%. The average protein contents of the three lines (HHF7-3-10, HHF7-6-6, and HHF7-6-10) from the Huang-Huai Valleys ranged from 42.3% to 43.5%. These six resistant lines, which have increased seed oil or protein contents, could be used as resistant cultivars against SCN or advanced donor parents in soybean breeding programs.

## 1 Introduction

Soybean (*Glycine max*) is one of the most important crops that provides a sustainable source of protein and oil worldwide. However, soybean yields continue to decrease to unprecedented levels with losses of approximately 800 million USD in China (Peng et al., 2021) and more than 1 billion USD in the United States annually owing to the soybean cyst nematode (SCN) disease caused by *Heterodera glycines* (Koenning and Wrather, 2010). SCN has spread into the primary areas in which soybean is grown worldwide (Lin et al., 2022). Owing to the use of management techniques, such as crop rotation and the planting of varieties resistant to SCN, which are not commonly accepted by farmers, SCN resistance breeding is still at an early developmental stage, especially in China (Lian et al., 2022). One of the reasons is that most farmers may not be aware that their fields are infected with SCN, as there is no regular survey or service system in China where soil samples can be collected for SCN testing. Additionally, compared to popular local cultivars, the yield of SCN-resistant cultivars is still lower.

Although plants have evolved different mechanisms for resistance against SCN, resistance breeding is still challenging owing to the dynamic and evolving nature of host-pathogen interactions. This results in the continual emergence of virulent populations of pathogens that overcome formerly resistant crop varieties (Nelson et al., 2018). In fact, the field populations of SCN are genetically variable, and the dominant populations of SCN shift in the composition and complexity of their virulence. For example, the predominant race of SCN in the Huang-Huai Valleys, China, was race 2, HG types 1.2.5.6.7-/1.2.5. 7-, surveyed in 2010-2015 (Lian et al., 2016) compared with race 1 surveyed in 2001-2003 (Lu et al., 2006). The predominant race in Missouri, US, was race 2, surveyed in 2015-2016 (Howland et al., 2018), compared with race 1, surveyed in 2005 (Mitchum and Wrather, 2007). In the US, more than 95% of the commercial varieties rely on PI88788 as their source of resistance to SCN (Niblack et al., 2008), which has caused virulent populations of nematodes to develop over time owing to the long-term planting of PI88788-type resistance. Furthermore, a highly virulent population of SCN designated X12 was recently reported (Lian et al., 2017, 2019), which can successfully parasitize all types of resistant soybean germplasm tested, including the four indicator lines of the race scheme (Riggs and Schmitt, 1988), seven indicator lines of the HG type test (Niblack et al., 2002), the elite resistant germplasm ZDD2315, and PI567516C. All these observations indicate that the ecological environment has majorly influenced the evolution of virulence in nematodes. Therefore, it is imperative to identify and breed new genetic sources of resistance for sustainable agroecology and agricultural production to mitigate the mechanisms by which pathogens overcome the genes for plant resistance and minimize the accumulation of virulence genes.

Marker-assisted-selection (MAS) has been used to breed crops, such as maize (*Zea mays*), wheat (*Triticum aestivum*), and rice (*Oryzae sativa*), with speed and accuracy (Haque et al., 2023; Hu et al., 2023; Suvarna et al., 2023). Kompetitive allele specific PCR (KASP) markers in the soybean genome have been identified that are specific to the *rhg1* and *Rhg4* genes that are associated with phenotypic variations that are resistant to SCN (Shi et al., 2015; Kadam et al., 2016; Shaibu et al., 2022). In this study, six advanced breeding lines were generated using a single seed descent (SSD) approach and selected by genotyping and phenotyping for desirable agronomic traits in the field. All six lines were of the Peking-type resistance and resistant to multiple SCN races. Two resistant lines originated from Northeast China with a more than 22% seed oil content and three resistant lines from the Huang-Huai Valleys with a protein content of more than 42%. It is valuable to identify varieties resistant to SCN for the Chinese market and the potential to further improve soybean breeding using these advanced donor parents toward high-yielding varieties resistant to SCN. This is also an example of marker-assisted selection in plant breeding.

## 2 Results

### 2.1 Breeding advanced breeding lines

**Figure 1:**
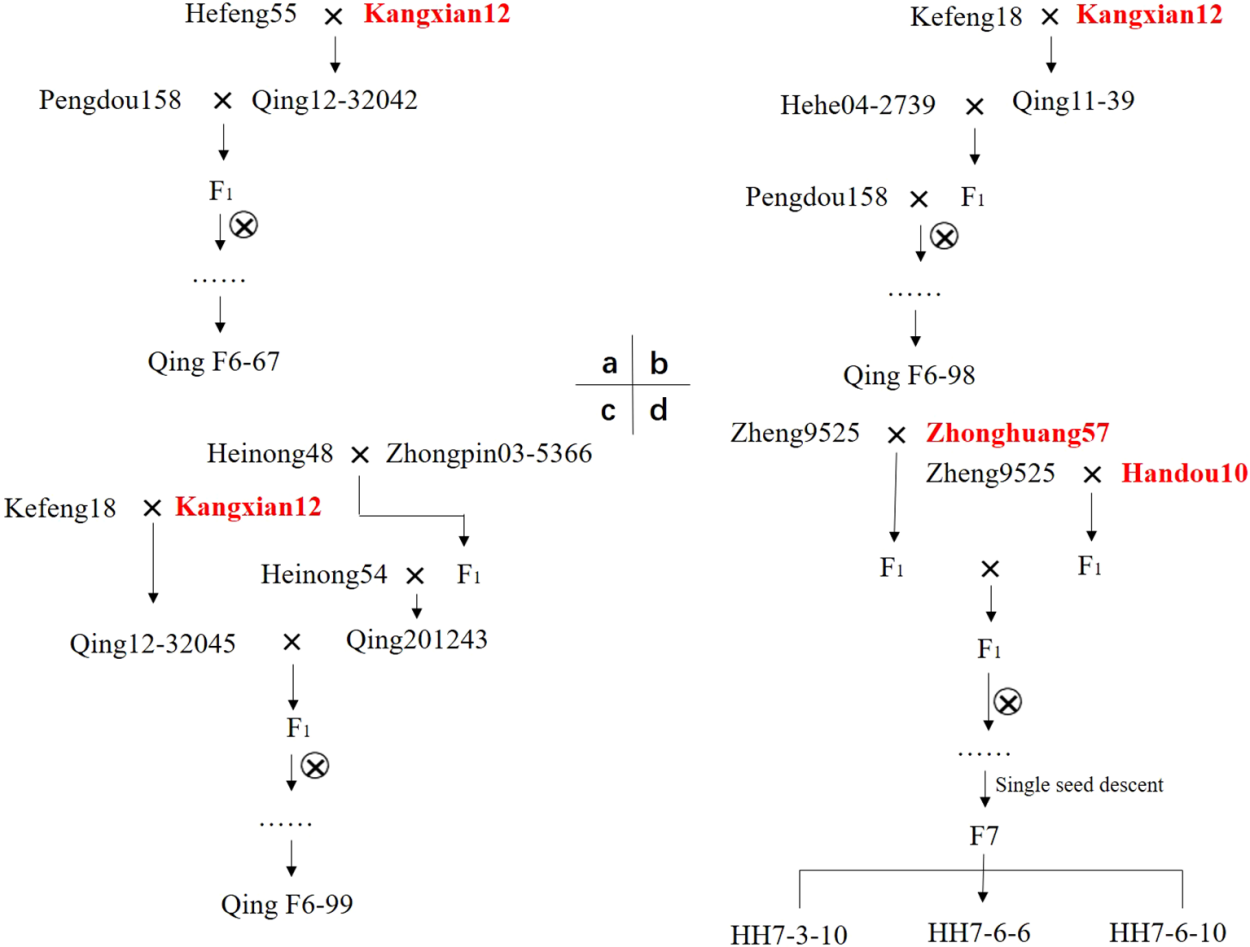
Breeding of the soybean cultivars QingF6-67, QingF6-98, QingF6-99, HHF7-3-10, HHF7-6-6, and HHF7-6-10 by a pedigree breeding method. Kangxian12, Zhonghuang57, and Handou10 are the SCN-resistant donor parents highlighted in bold red font. a, b, and c represent the breeding pedigree of QingF6-67, QingF6-98, and QingF6-99, respectively; d represents the breeding pedigree of HHF7-3-10, HHF7-6-6, and HHF7-6-10.

After 6 to 7 years of continuous self-pollination under the single-seed descent (SSD) method, subsequent molecular marker selection, SCN-infestation testing, and further agronomic trait evaluations in 2023, six advanced breeding lines, namely, QingF6-67, QingF6-98, QingF6-99, HHF7-3-10, HHF7-6-6, and HHF7-6-10, were developed. The pedigree breeding scheme is shown in **Figure 1**.

### 2.2 The primary agronomic traits of the six advanced breeding lines

**Figure 2:**
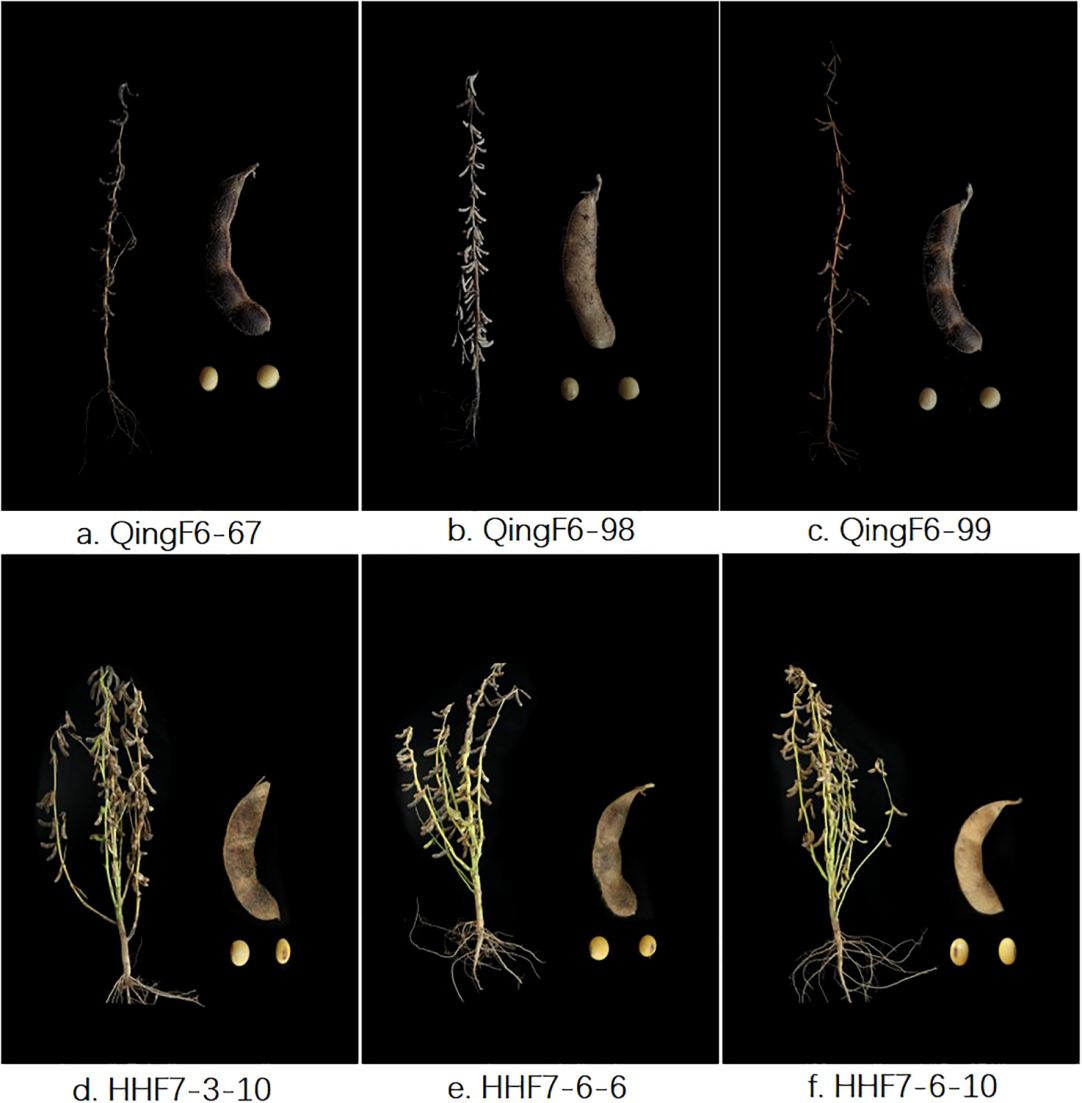
The six advanced breeding lines and pod and seed phenotypes at the maturity stage. Selected lines with erect types in growth, brown pods, and yellow seeds are shown. **(a–c)** are QingF6-67, QingF6-98, and QingF6-99, respectively, which were cultivated from Northeast China; **(d–f)** are HHF7-3-10, HHF7-6-6, and HHF7-6-10, respectively, which were cultivated from the Huang-Huai Valleys.

All selected advanced breeding lines in this study were erect types regarding growth and had yellow seeds. The key agronomic traits of QingF6-67, QingF6-98, and QingF6-99 were surveyed in northeast China, which had a height of 82.12 ± 3.11 cm, 77.41 ± 2.31 cm, and 106.66 ± 3.50 cm, respectively, and few branches on the mature plants. The 100-seed weights were 21.48 ± 0.75 g, 19.59 ± 0.85 g, and 21.1 ± 0.84 g, respectively, compared with 18.85 ± 0.64 g of the donor parent. They all had a yellow coat and hilum (**Figure 2**). HHF7-3-10, HHF7-6-6, and HHF7-6-10 were surveyed in the Huang-Huai Valleys and had a height of 80.02 ± 2.74 cm, 91.22 ± 3.79 cm, and 86.00 ± 2.63 cm, respectively, with four branches on the mature plants on average. The 100-seed weights were 21.60 ± 0.73 g, 18.91 ± 0.73 g, and 19.12 ± 0.47 g compared with 18.3 ± 0.50 g of the donor parent. They all had a yellow coat and brown hilum except for HHF7-3-10, which had a black hilum (**Figure 2**). Other agronomic traits, such as first pod height, node number, pod number per plant, and grain yield per plant, were also recorded (**Table 1**). Moderate branching, upright growth, and concentrated fruit distribution are key to increasing soybean yield, except for resistant disease.

**Table 1:** Agronomic traits of the selected six advanced breeding lines.

Name	Plant height (cm)	First pod height (cm)	Nodes number	Branch number	Seed number per plant	Grain yield per plant (g)	100-seed weight (g)	Seed coat color	Hilum color
Qing F6-67	82.12 ± 3.11	13.58 ± 2.51	13.90 ± 1.20	1.60 ± 1.07	113.40 ± 23.48	24.44 ± 5.13	21.48 ± 0.75	Yellow	Yellow
Qing F6-98	77.41 ± 2.31	10.29 ± 4.01	13.90 ± 1.20	1.10 ± 0.88	105.20 ± 19.25	20.61 ± 3.52	19.59 ± 0.85	Yellow	Yellow
Qing F6-99	106.66 ± 3.50	15.27 ± 4.92	16.10 ± 1.60	3.30 ± 1.57	101.30 ± 27.11	21.17 ± 5.83	21.1 ± 0.84	Yellow	Yellow
*Kangxian 12*	82.03 ± 5.92	7.75 ± 2.15	17.20 ± 0.79	3.20 ± 1.23	159.80 ± 46.43	29.18 ± 7.97	18.85 ± 0.64	Yellow	Black
LF7 3-10	80.02 ± 2.74	9.21 ± 3.43	15.92 ± 1.94	3.8 ± 0.57	118.19 ± 17.54	25.53 ± 4.81	21.60 ± 0.73	Yellow	black
*LF7 6-6*	91.22 ± 3.79	8.86 ± 2.47	16.75 ± 1.23	4.10 ± 1.84	115.22 ± 15.99	21.79 ± 6.56	18.91 ± 0.73	Yellow	brown
*LF7 6-10*	86.00 ± 2.63	9.05 ± 3.89	15.30 ± 1.04	3.95 ± 0.82	122.47 ± 23.22	23.42 ± 4.77	19.12 ± 0.47	Yellow	brown
*Zhonghuang 57*	54.1 ± 2.44	6.51 ± 3.58	12.70 ± 1.27	4.30 ± 0.89	97.30 ± 15.40	17.61 ± 4.52	18.30 ± 0.50	Yellow	Yellow
*Handou 10*	119 ± 4.23	15.00 ± 3.12	21.50 ± 0.89	2.30 ± 1.17	120.56 ± 18.47	22.42 ± 2.89	18.60 ± 0.76	Yellow	Yellow

### 2.3 Contents of protein, oil, and isoflavones in the seeds

The contents of protein, fat, and isoflavones in the seeds of the six advanced breeding lines and three resistance donor parents were measured (**Table 2**). Oil content ranged from 19.96% to 22.74% in QingF6-67, QingF6-98, and QingF6-99, compared with 19.61% in the donor parent Kangxian 12. The protein content ranged from 42.3% to 43.5% in HHF7-3-10, HHF7-6-6, and HHF7-6-10 compared with 41.7% in the donor parent Zhonghuang 57 and 41.3% in the donor parent Handou10. The six primary components of soybean isoflavones including daidzin, daidzein, and genistein, were also measured. Overall, the total soybean isoflavone content was 1,071.19 mg/kg, 856.72 mg/kg, and 700 mg/kg in QingF6-67, QingF6-98, and QingF6-99, respectively, compared with 692.72 mg/kg in the donor parent Kangxian 12. This shows that the isoflavone content increased in each advanced breeding line. The soybean isoflavone content was 603.31 mg/kg, 971.86 mg/kg, and 724.82 mg/kg in HHF7-3-10, HHF7-6-6, and HHF7-6-10, respectively, compared with 529.65 mg/kg in the donor parent Zhonghuang57 and 1,074.56 mg/kg in the donor parent Handou10.

**Table 2:** The quality of protein, oil, and soybean isoflavones in the six advanced breeding lines.

Name	Protein content %	Oil content %	Daidzin mg/kg	Glycitin mg/kg	Genistin mg/kg	Daidzein mg/kg	Glycitein mg/kg	Genistein mg/kg	Total soybean isoflavone
QingF7-67	36.2 ± 0.07	22.74 ± 0.06	500 ± 31.11	67.6 ± 1.98	486 ± 31.11	2.93 ± 0.03	9.5 ± 0.63	5.16 ± 0.12	1071.19 ± 64.74
QingF7-98	35.4 ± 0.07	22.23 ± 0.21	306 ± 14.14	71.7 ± 1.84	466 ± 19.8	1.38 ± 0.04	8.18 ± 0.15	3.46 ± 0.03	856.72 ± 35.86
QingF7-99	38.9 ± 0.14	19.96 ± 0.06	272 ± 13.44	77.1 ± 3.25	338 ± 13.44	2.08 ± 0.02	6.64 ± 0.28	4.18 ± 0.08	700 ± 30.34
*Kangxian 12*	38.6 ± 0.07	19.61 ± 0.1	274 ± 10.61	74.4 ± 3.04	331 ± 7.07	2.71 ± 0	6.52 ± 0.3	4.09 ± 0.25	692.72 ± 14.09
HHF7-3-10	43.5 ± 0	18.96 ± 0.12	266 ± 8.49	51 ± 1.34	268 ± 16.26	5.18 ± 0.35	6.27 ± 0.27	6.86 ± 0.28	603.31 ± 22.51
HHF7-6-6	42.3 ± 0	17.33 ± 0.06	385 ± 18.38	60.7 ± 3.54	502 ± 33.23	7.98 ± 0.23	11.5 ± 0.14	4.68 ± 0.16	971.86 ± 48.3
HHF7-6-10	42.4 ± 0.07	16.82 ± 0.05	313 ± 7.07	58.2 ± 0.14	337 ± 11.31	3.94 ± 0.03	7.84 ± 0.21	4.84 ± 0.16	724.82 ± 17.9
*Zhonghuang 57*	41.7 ± 0.07	20.73 ± 0.11	185 ± 5.66	49.2 ± 0.35	265 ± 4.24	11.4 ± 0.28	8.25 ± 0.21	10.8 ± 0.07	529.65 ± 9.69
*Handou 10*	41.3 ± 0.14	20.84 ± 0.12	395 ± 9.9	192 ± 0.71	454 ± 13.44	6.26 ± 0.29	18.6 ± 0.42	8.7 ± 0.02	1074.56 ± 23.89

### 2.4 KASP molecular marker analysis

KASP assays were performed for all the single-nucleotide polymorphisms (SNPs) in the advanced breeding lines at the *rhg1* and *Rhg4* loci using the soybean lines with known reactions to SCN as a control (**Table 3**; **Supplementary Table S1**). GSM383 is an SNP functional marker to differentiate Peking-source with a G allele in the *Rhg1* loci and PI88788-sources with a C allele in the *Rhg1* loci for resistance. All six advanced lines and the control lines Peking and PI437654 have similar haplotypes. All are the Peking/PI437654-source marker haplotype for the *rhg1* and *Rhg4* resistance alleles (GSM381=G, GSM383=G, and GSM191=G).

**Table 3:** Marker haplotypes at SCN loci *rhg1* and *Rhg4*, race testing phenotypes, and the corresponding resistance type of six soybean lines and four known soybean reference lines.

Name	Markers at locus *rhg1*		Marker at locus *Rhg4*	Resistance reaction to multiple SCN races				Resistance type according to marker haplotypes at *rhg1* and *Rhg4*	Resistance type according to race testing
	GSM381 (Gm18: 645407) #1	GSM383 (Gm18: 1643660)	GSM191 (Gm08: 8361148)	Race1 #2	Race2	Race3	Race5	Same haplotype as the reference line	FI pattern comparable to the reference line
Lee	T	C	C	S	S	S	S	S	S
Peking	G	G	G	R	S	R	R	Peking	Peking
PI 88788	G	C	C	S	MR	R	MR	PI 88788	PI 88788
PI 437654	G	G	G	R	R	R	R	Peking	PI 437654
QingF7-67	G	G	G	R	S	R	R	Peking	Peking
QingF7-98	G	G	G	R	S	R	R	Peking	Peking
QingF7-99	G	G	G	R	MS	R	R	Peking	Peking
HHF7-3-10	G	G	G	MR	MR	R	R	Peking	Peking
HHF7-6-6	G	G	G	MR	S	R	R	Peking	Peking
HHF7-6-10	G	G	G	MR	MS	R	R	Peking	Peking

#1: Marker name and PCR according to Shi et al. (2015). The number represents the genome coordinate based on the Database: Gmax_275_v2.0.softmasked.

#2: Resistance level definition according to female index: R, resistant; MR, moderately resistant; MS, moderately sensitive; S, sensitive.

### 2.5 Evaluation of the resistance to SCN using races 1, 2, 3, and 5

SCN resistance phenotypes of the advanced breeding lines with a resistance-associated genotype, including 28 that originated from the Huang-Huai Valleys and 19 that originated from Northeast China, were evaluated in detail by inoculating races 1, 2, 3, and 5 in separate bioassays (**Table 4**; **Supplementary Table S2**). Only the data of the six lines that were finally selected are shown here. All six lines were found to be resistant to races 1, 3, and 5. Three lines from Northeast China were resistant to race 1 with a female index (FI) that ranged from 0.5 to 2, and three lines from the Huang-Huai Valleys were moderately resistant to race 1 with an FI that ranged from 12 to 13.3. All six lines were resistant to races 3 and 5, while those that were sensitive or moderately sensitive to race 2 had varied FI values. Briefly, QingF6-67, QingF6-98, QingF6-99, HHF7-3-10, HHF7-6-6, and HHF7-6-10 were resistant to races 1, 3, and 5 (resistance types like Peking). The SNPs in *rhg1* and *Rhg4* are associated with this type of SCN resistance. All the sources used as resistant parents in this study exhibit Peking-type resistance, with the phenotypic and genotypic analysis results of Zhonghuang57 and Handou10 published by Lian et al. (2023), and of Kangxian12 published by Chen et al. (2023).

**Table 4:** SCN race test of the cultivars against the four most common soybean cyst nematode races.

Cultivar/Accession name	Race 1			Race 2			Race 3			Race 5		
	Average number of cysts per plant #1	Female index #2	Resistance level #3	Average number of cysts per plant	Female index	Resistance level	Average number of cysts per plant	Female index	Resistance level	Average number of cysts per plant	Female index	Resistance level
Lee 68	268.5 ± 9.1(262-275)	/	S	204.25 ± 66.6(125-273)	/	S	214 ± 98.9(144-284)	/	S	420 ± 81.5(293-497)	/	S
QingF7-67	5.5 ± 7.7(0-11)	2	R	382.4 ± 77.4(285-458)	187.1	S	6 ± 1.4(5-7)	2.8	R	15.4 ± 6.5(8-25)	3.7	R
QingF7-98	4.5 ± 2.1(3-6)	1.6	R	496.4 ± 137(298-645)	242.9	S	2.5 ± 0.7(2-3)	1.1	R	8.8 ± 4.6(3-15)	2.1	R
QingF7-99	1.5 ± 0.7(1-2)	0.5	R	82.2 ± 41.8(39-140)	40.2	MS	0.5 ± 0.7(0-1)	0.2	R	0.4 ± 0.8(0-2)	0.1	R
Lee 68	89.6 ± 107.2(8-272)	/	S	181.8 ± 104(38-283)	/	S	65.6 ± 53.2(12-147)	/	S	364.4 ± 230.5(204-771)	/	S
HHF7-3-10	12 ± 7.9(3-24)	13.3	MR	50.4 ± 30.7(15-85)	27.7	MR	1 ± 1.7(0-4)	1.5	R	22.4 ± 20.2(9-56)	6.1	R
HHF7-6-6	10.8 ± 4.7(6-18)	12	MR	140.2 ± 97.8(31-272)	77.1	S	4.6 ± 4.6(0-11)	7	R	1.6 ± 1.5(0-4)	0.4	R
HHF7-6-10	11.6 ± 12.4(3-32)	12.9	MR	92.75 ± 65.4(25-175)	51	MS	5.2 ± 3.4(1-10)	7.9	R	2 ± 1.4(0-4)	0.5	R

#1: The average number of cysts per plant ± standard deviation and minimum–maximum number of cysts per plant in parenthesis.

#2: The female index (FI) is calculated as the mean number of cysts for each line relative to the mean number of cysts per plant of the susceptible control line Lee68, multiplied by 100. Bioassay was replicated three times with five plants per race testing for each line.

#3: Resistance level definition: FI ≤ 10 = R (resistant, R); 10 <FI ≤30 = MR (moderately resistant, MR); 30< FI ≤60 = MS (moderately sensitive, MS); FI >60 = S (sensitive, S).

## 3 Discussion

Plant diseases are responsible for substantial crop losses each year and threaten global food security and agricultural sustainability (Koenning and Wrather, 2010). Harnessing the resistance of hosts offers an effective and reliable method compared with the use of pesticides to prevent losses in yield (Lin et al., 2022). The generation of advanced breeding lines that contribute to resistance breeding for its agronomic traits is more conducive to increasing yield, quality, and agronomic adaptation than wild relatives, landraces, or other germplasm types.

In this study, breeding lines and donor parents were surveyed in different environments. The reasons are as follows. First, there are two principal soybean-producing regions in China, Northeast China and the Huang-Huai Valleys, and SCN infestation is widespread in both regions. However, the predominant races differ, with races 1 and 3 majorly distributed in Northeast China (Hua et al., 2018) with race 2 more prevalent in the Huang-Huai Valleys (Lian et al., 2016). Second, Northeast China and the Huang-Huai Valleys are classified geographically into distinct maturity groups. Northeast China is classified into maturity group I, while the Huang-Huai Valleys is in maturity group III. Third, the resistant parent of Kangxian12 cultivated in Northeast China exhibited resistance to races 1 and 3 but with low 100-seed weights, while Zhonghuang57 cultivated in Huang-Huai Valley exhibited resistance to races 1, 2, and 4 but had a multi-branch architecture. Therefore, in order to breed advanced donor parents towards high-yield or varieties with an ideal plant type, resistant to SCN, the advanced lines and donor parents were surveyed in different environments.

Six advanced breeding lines were finally generated and genotyped for the known SCN resistance genes *rhg1* and *Rhg4* (Concibido et al., 2004; Cook et al., 2012; Liu et al., 2017). All six advanced breeding lines had the Peking-type molecular marker haplotype of SCN resistance and exhibited resistance against races 1, 3, and 5 in environmentally controlled SCN bioassays (**Tables 3**, **4**). The six advanced breeding lines were also phenotyped for resistance against SCN race 2 and exhibited moderately susceptible or susceptible phenotypes. It is known that most resistant germplasms exhibit tight linkage to undesirable agronomic characteristics, such as black seed coats, wild or semi-wild types, shattering pods, and low seed yields and quality. Among the types of germplasm that are resistant to SCN, few have been used successfully to breed for resistance to SCN to date. It has been reported that more than 90% of the commercially available soybean cultivars in the US that are resistant to SCN are derived from PI 88788 (Niblack et al., 2002; Van and McHale, 2017; Wang et al., 2022). The primary reason for this is that PI 88788 harbors desirable agronomic characteristics. In this study, the six selected lines harbor Peking-type resistance haplotypes carrying both *rhg1* and *Rhg4* alleles, which confer durable resistance by reducing reliance on single-locus genes such as PI88788-type resistance (*rhg1*). This is particularly significant because the evolution of virulent nematode populations has diminished the effectiveness of many single-gene-resistant cultivars over time.

Protein and oil are the two principal constituents of seeds that make soybean an important crop. For decades, soybean breeding programs have aimed to increase the accumulation of oil and protein contents in soybean seeds (Van and McHale, 2017; Liu et al., 2022). Generally, the oil content in commercial varieties in China varies between 18% and 22% and the protein content between 36% and 42% (Xu et al., 2015; Qin et al., 2022). Considering that the protein and oil contents in soybean are seldom more than 40% and 20%, respectively, both QingF6-67 and QingF6-98 are considered high-oil cultivars, and HHF7-3-10, HHF7-6-6, HHF7-6-10 are considered high-protein cultivars. All six lines developed in this study exhibit resistance to multiple SCN races (1, 3, and 5) and possess favorable agronomic traits, including yellow seed coats, reduced shattering, higher 100-seed weights (18.91g to 21.6g), and enhanced seed fat or protein content. QingF6-67 and QingF6-98 are high-oil-content lines with seed fat contents of 22.74% and 22.23%, respectively, while HHF7-3-10, HHF7-6-6, and HHF7-6-10 are high-protein-content lines with protein contents exceeding 42%. Their broad resistance to globally prevalent SCN races, combined with superior agronomic qualities, makes these lines ideal candidates for both direct cultivation and use as advanced donor parents in soybean breeding programs aimed at improving yield and resilience in future breeding practices. SCN race 2 is the predominant race in the Huang-Huai Valleys (Lian et al., 2016). Thus, cultivars exhibiting resistance to SCN race 2 are increasingly essential. Unfortunately, the six lines developed in this study exhibit moderate susceptibility to SCN race 2.

Isoflavones potentially benefit human health and play pivotal roles in rhizobia-legume symbiosis and defense responses, and they are a class of flavonoids that are primarily found in legumes (Malla et al., 2020; Wu et al., 2020; Sohn et al., 2021). The total content of soybean isoflavones, including daidzin, daidzein, genistein, daidzein, daidzein, and genistein, varied from 603.31 to 1,071.19 mg/kg in the six advanced breeding lines. These isoflavones contribute to antioxidant activity, help prevent plant-related diseases, and positively affect plant robustness.

Therefore, using these advanced breeding lines as donor parents enhances the likelihood of preserving the resistance of soybean to races 1, 3, and 5, while maintaining or potentially enhancing the quality of protein and oil. Multiplex SCN resistance is also a valuable preventive measure to prevent the breakdown of resistance and the evolutionary dynamics of the SCN populations.

In conclusion, the six advanced breeding lines included here have proven to be of agronomic value with high contents of oil or protein and favorable seed coats, plant types, and heights, which makes them more easily accessible for modern breeding. Moreover, QingF6-67 and QingF6-98 are high-oil breeding lines with oil contents that exceed 20%, 22.74%, and 22.23%, respectively. QingF6-67 also has a high content of isoflavones that reach 1,071.19 mg/kg. HHF7-3-10, HHF7-6-6, and HHF7-6-10 are high protein breeding lines with protein contents that exceed 42 g/100 g: 43.5 g/100 g, 42.3 g/100 g, and 42.4 g/100 g, respectively.

## 4 Materials and methods

### 4.1 Plant materials and sources of SCN

Five soybean cultivars that are members of maturity group I, namely, Kangxian12 (resistant parent), Hefeng55, Kenfeng18, Heinong48, and Zhongpin03-5366, and three soybean cultivars that are primarily members of maturity group III, namely, Zhonghuang57 (resistant parent), Handou10 (resistant parent), and Zheng9525, were used as parents in this study. Additionally, Lee, Lee 68, PI 437654, Peking, and PI 88788 were used as the controls in the bioassay evaluations for SCN resistance and KASP analyses. In particular, of the donor parents used in this study, Kangxian12 is resistant to races 1, 3, and 5 but has a low 100-seed weight and oil content. Zhonghuang57 exhibits resistance to races 1-5 but possesses less desirable characteristics, including improper plant height and excessive branching. Handou10 exhibits resistance to races 1, 3, and 5 but it is limited to spring planting in the central and northern parts of Hebei Province. Lee is susceptible to SCN and was used as the susceptible check. The HG Type 2.5.7 (race 1), HG Type 1.2.5.7 (race 2), HG Type 7 (race 3), and HG Type 2.5.7 (race 5) SCNs used in the study were isolated from single cysts.

### 4.2 Breeding the advanced breeding lines

QingF6-67, QingF6-98, and QingF6-99 have the same resistant parent, designated Kangxina12, and were developed in Northeast China at Heilongjiang Academy of Agricultural Sciences (Harbin, China). The F1 seeds were continuously self-fertilized until the sixth generation using SSD. HHF7-3-10, HHF7-6-6, and HHF7-6-10 were crossed between the F1 from Zheng9525 crossed with Zhonghuang57 and F1 from Zheng9525 crossed with Handou10 in the HeNan Academy of Agricultural Sciences (Zhengzhou, China). These F1 seeds from two-way hybrids obtained in 2016 were self-crossed continuously until the seventh generation using SSD. The F6/F7 plants with favorable agronomic traits were selected and planted into rows for larger seed harvests for marker, bioassay, and further evaluations of their agronomic traits in 2022. In total, 48 single plants were harvested from the F6 generation which originated from Northeast China, and 51 single plants from the F7 generation which originated from the Huang-Huai Valleys. A genotype analysis to evaluate the SCN resistance loci using F6:7 or F7:8 seeds was conducted, and a bioassay to subsequently evaluate the resistance to SCN resistance was conducted in a controlled climate room by inoculating 2,500 eggs for each plant tested. Only the lines that contained the SCN resistance loci were examined in the bioassay, as determined by the genotype results, which resulted in bioassay tests of 28 out of the 51 lines cultivated in the Huang-Huai Valleys and 19 out of the 48 lines cultivated in Northeast China. The key agronomic traits of selected advanced breeding lines were evaluated separately at two locations, i.e., the Qiqihar Soybean Breeding Experimental Center of Heilongjiang (19 lines tested, N47°14′08”, E123°40′33”) and the Huang-Huai National Regional Trial Experimental Center (28 lines tested, N35°21′1″, E113°43′1″). Each line was sown in a plot of size 6.4 m^2^ with 4 meters in each row and four rows. Plant height (cm), first pod height (cm), node number, branch number, pod number per plant, grain yield per plant (g), 100-seed weight (g), seed coat color, and hilum color were recorded. All determined agronomic traits for each line were based on average values of ten randomly selected plants. The recommended crop production practices were followed throughout the experiment to reach the maximum yield potential of the crop. In 2023, abundant rainfall led to severe lodging of soybeans. Finally, six lines were selected by comprehensively considering both their resistance and agronomic traits.

### 4.3 Analysis of the SCN resistance loci in rhg1/Rhg4 using molecular markers

For marker analyses, the DNA was isolated from the fresh leaves of 2-week-old plants using magnetic beads (MagAttract 96 DNA Plant Core Kit; Qiagen, Germany). Its concentration was determined by a NanoDrop 2000 ultra micro-UV spectrophotometer (Thermo Fisher Scientific, Waltham, MA, USA), and 5 ng of DNA was used to detect the integrity of DNA in 1% agarose gel electrophoresis and stored at -20 °C. The primers of GSM381 (Gm18: 645407), GSM383 (Gm18: 1643660), and GSM191 (Gm08: 8361148) were synthesized using the KASP marker sequence described by Shi et al. (2015). The cycling conditions were used as described by KBiosciences (Herts, UK), and the fluorescent end-point genotyping method was conducted using a Roche LightCycler instrument (Roche, Basel, Switzerland). The plants tested were genotyped with the functional SNP markers (two for *rhg1* and one for the *Rhg4* locus). GSM191 identified the resistant (G) and sensitive (C) loci in the *Rhg4* allele, and GSM381 discriminates between resistant and susceptible *Rhg1* alleles. In addition, GSM383 is a functional SNP that can differentiate Peking(G) and PI88788(C) loci in the *Rhg1* allele.

### 4.4 Protein/oil extraction and determination in soybean seeds

The protein contents in soybean seeds were determined according to the modified Kjeldahl method (Bradstreet, 1954). Briefly, 200 mg heat-dried fine powder was weighed into a micro Kjeldahl digestion unit and digested with 15 mL H_2_SO_4_ and composite catalyst (CuSO_4_ and K_2_SO_4_) overnight. The samples were then incubated under the following successive temperature conditions: 160°C, 15 min; 220°C, 30 min; 350°C, 30 min; 450°C, 120 min. Nitrogen content was determined using an automatic Kjeldahl apparatus (KjeltecTM 8400, FOSS, Denmark). Protein percentage was calculated by percentage N multiplied by the factor 6.25.

The fatty acid content in soybean seeds was determined using the method described by Fan et al. (2015) with minor modifications. Briefly, dried mature seeds were ground into a fine powder and then transferred into a 15 mL glass tube filled with 2 mL extraction buffer (chloroform: isopropanol, 2:1). Subsequently, they were treated with 2 mL 1% sulfate in methanol (v/v) at 80°C for 1 h and then extracted for FAME determination with 3 mL hexane and 1 mL 0.9% (w/v) NaCl using 1 mL hexane phase for FAME detection on a gas chromatography equipped with a DB-23 column (Agilent, CA, USA). The samples were then incubated under the following successive temperature conditions: 120°C for 5 min; increased by 4°C min^-1^ to 190°C and held for 12 min; continued to increase to 210°C at 2.5°C min^-1^ and maintained for 10 min at the final temperature. Furthermore, 1 µL of each sample was injected and detected by a gas chromatography-flame ionization detector (7890A, Agilent, USA) at 280°C. The absolute difference between two independent measurement results obtained under identical conditions should not exceed 10% of the arithmetic mean.

### 4.5 Isoflavone extraction and determination in soybean seeds

Soybean seed isoflavones were extracted and characterized following the protocol from Zhang et al. (2007). Briefly, seeds were ground into powder, which was then sifted through a 40-mesh screen. Degreasing was performed with petroleum ether at 65°C for 2 h, and then samples were dried at 37°C to constant weight. Furthermore, 250 mg of skimmed powder was dissolved in 10 mL of 80% methanol at room temperature for 2 h, and the mixture was subsequently distilled at 80°C for 12 h. After centrifugation at 12,000 rpm for 15 min, 1 mL of the supernatant solution was filtered into a separate HPLC vial for isoflavones determination. The standard solutions of six major isoflavone components (daidzin, glycitin, genistin, daidzein, glycitein, and genistein) with purity over 98% (Sigma-Aldrich, USA) were dissolved in 100 mg/L of methanol preparations to establish the calibration curves. Employing high-performance liquid chromatography (HPLC, Shimadzu, Kyoto Prefecture, Japan), isoflavone components in seeds were identified and quantified according to the retention times and peak areas of six standard isoflavone solutions. The sum of the contents of these six major components represented the total isoflavone contents. All samples were analyzed in triplicate.

### 4.6 Nematode resistance test

Soybean cyst nematode populations of races 1, 2, 3, and 5 were propagated in a controlled climate room under non-sterile conditions on susceptible SCN plants to rear sufficient numbers of eggs. The fully developed brown cysts from the roots were harvested and prepared for inoculation. For environmentally controlled resistance bioassays, only the accessions that harbored the resistant alleles were evaluated for their resistance to SCN following a well-established greenhouse bioassay at the Henan Academy of Agricultural Sciences. Five plants were tested for each line, indicator lines of the Riggs model, including Pickett, Peking, PI 88788, and PI 90763 (Riggs and Schmitt, 1988), and the susceptible check (Lee 68) were arranged. Seedlings that had germinated after 3 days at 25°C were transplanted into disposable plastic cups and filled with sterile soil that was sterilized at 150°C for 1.5 h. Furthermore, 3 or 4 days after transplantation (one seedling/cup), the eggs of races 1, 2, 3, or 5 were inoculated on the roots. Approximately 2,500 fresh eggs were inoculated on each plant using a peristaltic pump. The experiments were maintained at 28°C/25°C (day/night) and watered daily. At 25–30 days post-inoculation, the cysts were collected by carefully washing the roots of each plant and imaged using a fluorescence-based imaging system developed by the Soybean Research Group of Henan Academy of Agricultural Sciences. The cysts were then counted, and the FI was calculated using the percentage of the mean number of cysts per plant of each line relative to the mean number of cysts per plant of the susceptible control line ‘Lee’ to evaluate the resistance level to SCN.

## Data Availability

The original contributions presented in the study are included in the article/**Supplementary Material**. Further inquiries can be directed to the corresponding authors.

## Supplementary material

Supplementary Table 1 Marker haplotypes at SCN-resistant loci *rhg1* and *Rhg4* in selected single plants. Numbers with * denote the lines are selected for further SCN bioassay.

Supplementary Table 2 SCN race test results of the cultivars with resistant haplotypes at SCN-resistant loci *rhg1* and *Rhg4*.
